# Navigating the Cancer Journey Using Web-Based Information: Grounded Theory Emerging From the Lived Experience of Cancer Patients and Informal Caregivers With Implications for Web-Based Content Design

**DOI:** 10.2196/41740

**Published:** 2023-05-17

**Authors:** Maclean Thiessen, Shelly Raffin Bouchal, Patricia A Tang, Shane Sinclair

**Affiliations:** 1 Department of Internal Medicine Rady Faculty of Health Sciences University of Manitoba Winnipeg, MB Canada; 2 Faculty of Nursing University of Calgary Calgary, AB Canada; 3 Department of Oncology Cumming School of Medicine University of Calgary Calgary, AB Canada; 4 Compassion Research Lab Faculty of Nursing University of Calgary Calgary, AB Canada

**Keywords:** health information behavior, neoplasm, theory, internet, information needs, adults

## Abstract

**Background:**

The internet is an important source of information for many informal caregivers and patients living with cancer. A better understanding of how individuals use the internet to meet their informational needs is important for guiding intervention development.

**Objective:**

The objectives of this study were to develop a theory describing why individuals living with cancer use the internet to find information, characterize the challenges faced with existing web-based content, and provide recommendations for web-based content design.

**Methods:**

Adults (≥18 years) with a history of being patients with cancer or informal caregivers were recruited from Alberta, Canada. After providing informed consent, participants were engaged through digitally recorded one-on-one semistructured interviews, focus groups, a web-based discussion board, and emails. Classic grounded theory guided the study procedures.

**Results:**

A total of 21 participants took part in 23 one-on-one interviews and 5 focus groups. The mean age was 53 (SD 15.3) years. Breast, gynecological, and hematological cancers were the most common cancer types (4/21, 19% each). In total, 67% (14/21) of patients, 29% (6/21) of informal caregivers, and 5% (1/21) of individuals reporting both roles participated. Participants experienced many new challenges in their cancer journey and used the internet to become better oriented to them. For each challenge, internet searching attempted to address one or more of 3 key orientation questions: why the challenge was happening, what to expect, and options for managing it. Better orientation resulted in improved physical and psychosocial well-being. Content that was well laid out, concise, free of distractions, and that addressed the key orientation questions was identified as the most helpful in assisting with orientation. Creators of web-based content are encouraged to 1) clearly identify the cancer challenge and population the content is addressing, as well as the presence of any potentially distressing information; 2) provide versions of the content in different formats, including printer-friendly, audio, video, and alternative languages; 3) state who created the content, including the individuals, organizations, and processes involved; 4) place hyperlinks after the key orientation questions have been addressed; and 5) ensure that the content is optimized for discovery by search engines (ie, Google).

**Conclusions:**

Web-based content plays an essential role for many living with cancer. Clinicians are encouraged to take active steps to help patients and informal caregivers find web-based content that meets their informational needs. Content creators also have a responsibility to ensure that the content they create assists and does not hinder those navigating the cancer journey. Research is needed to better understand the many challenges that individuals living with cancer face, including how they are temporally related. In addition, how to optimize web-based content for specific cancer challenges and populations should be considered an important area for future research.

## Introduction

### Background

An information need is an individual’s recognition that their knowledge is inadequate to satisfy their goals [1]. Most of those living with cancer experience unmet information needs at some point [2-6]. In the curative intent setting, information needs have been identified as the most commonly unmet supportive care need [7], with a prevalence exceeding 50% [8]. In the noncurative intent setting, similar findings have been identified, with information needs consistently being one of the most common and important unmet supportive care needs [9]. For informal caregivers, including friends and family supporting a patient, information needs are just as important and likely to go unmet [10]. These findings are not limited to a few studies as the importance of information and the high prevalence of unmet information needs in the populations affected by cancer have been well characterized in many studies, including across different cancer types [11] and points in the cancer journey [3,12,13]. Importantly, the range of information needs experienced during the cancer journey is vast. A recent review identified that the number of distinct information needs characterized in the literature totaled 1709 [5]. The authors were able to organize these needs into 17 distinct categories and 119 subcategories ranging from treatment-related to financial and legal information [5].

Addressing the information needs of those living with cancer, including patients and informal caregivers, should be prioritized by both clinicians and health care systems. From a health care system perspective, a systematic review explored the impact of decisional support for health care interventions on the costs of care [14]. A total of 7 studies were included, with decisional support being provided primarily through information sharing interventions delivered via DVDs, booklets, web-based content, videotapes, and coaching. The review identified that the information interventions were associated with decreased costs of health care delivery, including reduced treatment use rates [14]. Despite not including studies in the cancer context, these findings can be extrapolated. For instance, a systematic review of shared decision-making in the lung cancer context demonstrated that shared decision-making resulted in decreased emergency room visits and a reduction in the amount of chemotherapy received [15], presumably resulting in decreased health care resource use and costs.

In terms of clinical consequences, unmet information needs have been shown to be associated with negative outcomes in the short and long term. A systematic review explored the link between information and physical and psychosocial outcomes in patients with cancer [16]. This study found that information provision, quality of information, and satisfaction with the information provided were positively associated with health-related quality of life and physical well-being and negatively associated with anxiety and depression [16]. Similar findings were identified in another systematic review focusing on the unmet care needs of both patients and informal caregivers in the advanced cancer setting [10]. For patients, unmet needs related to information provision, including communication with health care providers and specific information needs, were identified to be associated with increased symptom burden and distress [17]. For informal caregivers, the review did not specifically explore how information needs and provision were associated with physical or psychosocial well-being. However, a study included in the review identified that increased caregiver information needs were associated with increased fatigue (*P*=.005) [18].

The relationship between information and the physical and emotional well-being of patients and caregivers is, at least to some extent, causal. Support for this comes from both the theoretical and empirical literature. From a theoretical perspective, information plays a key role in coping with stress [19,20]. According to stress and coping theory [20], individuals engage in 2 types of coping when confronted with a new challenge: problem-based and emotional-based coping. Information can assist with both as it can help individuals decrease uncertainty about what is to come, resulting in decreased anxiety, and help individuals plan what action to take to promote an outcome that is in line with their personal goals. Through this theoretical lens [19-21], the published literature demonstrating a statistically correlated relationship between unmet information needs and higher levels of depression, anxiety, and increased psychosocial complaints [10,16,22] supports the important role that information plays in assisting with emotional coping.

The literature also provides support for information as a key part of problem-based coping. For instance, a quasi-experimental study [23] evaluated the impact of an educational intervention on the side effects of chemotherapy. Compared with usual care, participants in the experimental group received 3 personalized educational sessions focusing on the self-management of chemotherapy side effects. Participants in the experimental group experienced significantly less (ie, *P*<.05) nausea, constipation, pain, mouth sores, weight change, fatigue, and difficulty sleeping [23]. Information has also been shown to help individuals navigate the impact of the cancer journey on the aspects of their lives outside of being patients or informal caregivers by supporting them in coordinating their non–cancer-related social roles (eg, being employees, parents, and friends) around the demands of the cancer journey [24,25].

### The Internet and the Cancer Journey

Multiple studies have demonstrated that the internet is a key resource for those living with cancer. In a Swedish survey study of patients with cancer (N=282), 76.2% of the respondents reported using the internet to find cancer-related information after their diagnosis [26]. Another survey of patients with advanced breast cancer reported that 83% of the respondents used the internet to find information related to their diagnosis and to assist with navigating the cancer journey on a daily basis [27]. Other studies have identified that high rates of internet use are likely related to the fact that, unlike health care providers, the internet is accessible around the clock, does not require an appointment or travel to the physician’s office, and affords the individual anonymity [28].

Although the internet plays an important role for many living with cancer, it is by no means a perfect source of information. An internet connection and appropriate device are required, and individuals may not be aware of or feel comfortable accessing web-based information [28]. For those who can access the internet, the content may be inaccurate, misleading, or a source of confusion and distress [17,29,30]. A recent review of web-based content from 48 websites for patients with cancer about depression used a validated tool to evaluate the quality of the content [31]. This review identified issues with accountability in 63% of the websites and readability in 54% and found that only 38% of the websites had been updated in the last 2 years, raising concerns about content accuracy. Another study found that the information patients need and what is available on the web are not always well aligned [32].

### A Gap in the Literature

Developing web-based content to assist individuals living with cancer is a complex challenge where theory, rigorously grounded in empirical data from the cancer context, has an important role to play. Theory facilitates the identification of important factors and variables for planning how interventions are deployed, predicting expected outcomes, and informing what should be measured to assess efficacy [33]. In addition, theory can evolve over time, being revised as newly discovered scientific findings emerge to better reflect the phenomena in question [34,35]. Finally, theory provides common conceptual ground, promoting collaboration among researchers and institutions and across disciplines [36]. In the context of evolving how those living with cancer are cared for, the importance of structuring the development and evaluation of any novel intervention on a robust theory grounded in the cancer context cannot be overstated.

Several theoretical conceptualizations addressing how individuals living with cancer have their informational needs met exist. Those by Freimuth et al [37] and Longo [38] are important to mention as they both describe the information-seeking behavior of those living with cancer and were developed from data collected in the cancer context. The health information acquisition model by Freimuth et al [37] was developed using data collected from the Cancer Information Service, a telephone-based information service developed by the National Cancer Institute in the United States [37]. Longo developed a theory of health of information behavior beginning with initial work involving interviews with patients with breast cancer [38]. Notably, although not cancer-specific, Wilson [21] incorporated the work of both Longo [38] and Freimuth et al [37] as well as many other theorists and researchers across a number of disciplines and synthesized a comprehensive multidisciplinary theory of information-seeking behavior.

The work of Wilson [21], Freimuth et al [37], and Longo [39] provides important insight into how individuals living with cancer seek information. Importantly, the representative models from Freimuth et al [37] and Wilson [21] identify that important cyclical feedback mechanisms exist between stimuli, or perceived information needs, and information seeking [40]. Both models incorporate a cost-benefit (or risk-reward) analysis performed by the information seeker to determine whether the potential benefits of searching for more information outweigh the anticipated costs [21,40], such as time, energy, and potential emotional distress [25]. An important question that follows from these models relates to how web-based content can be created to optimize the benefit for the end user while minimizing costs.

To work toward addressing this important question, a rigorously developed theory grounded in the cancer experience is needed. This theory needs to conceptualize the challenges that individuals face when they turn to the internet and what makes internet content useful for addressing these challenges. Such a theory would be useful for guiding content creators in creating web-based content to better meet the needs of those living with cancer.

### Study Objectives

This study was conducted to understand how to better support those living with cancer through web-based information. The objectives of this study were to develop theoretical conceptualizations of (1) the goals that individuals living with cancer are trying to achieve [41] when they use the internet to find information, (2) the challenges they face with existing content, and (3) web-based content design elements that would assist them in meeting their informational needs.

## Methods

### Recruitment

Research participants were recruited from emailing lists maintained by Alberta Health Services, including individuals living with cancer, as well as a cancer support clinic network. Recruitment posters were placed in clinical areas accessible to ambulatory patients at a major health center in Calgary, Alberta. Participants did not have a previous relationship with the researcher (MT). They were informed of the researcher’s professional practice as a medical oncologist in Manitoba, Canada, and that the research project was being conducted in conjunction with the researcher’s doctoral thesis work.

### Data Collection

After providing informed consent, participants completed a short intake survey (Multimedia Appendix 1) capturing demographics and characterizing their cancer journey (ie, cancer type, treatment intent, and role as patient or informal caregiver) and their interest in participating in one-on-one interviews and focus groups. They then received a study-specific username and password to facilitate anonymous participation in the study’s web-based discussion forum as well as email correspondence with the study lead (MT). Study activities included digitally recorded one-on-one semistructured interviews (via telephone or Zoom; Zoom Video Communications), focus groups (via Zoom), email correspondence, and participation in a private password-protected web-based discussion forum.

An initial interview guide (Multimedia Appendix 2) was developed by the authors that was modified as the study progressed in keeping with classic grounded theory methodology [35]. One-on-one interviews and focus groups were conducted with individuals selected to ensure that all emerging concepts reached saturation. This involved identifying individuals for study activities based on their responses to the intake questionnaire, availability, and what was known about them from their responses in earlier study activities (ie, from previous interviews, focus groups, emails, and web-based forum responses). As concepts emerged, in addition to being explored through interviews and focus groups, questions were posed to all participants through the private online discussion forum as well as through emails.

### Data Analysis

The data collected included field notes (generated by the researcher during the interviews and focus groups), transcripts generated from audio recordings of the interviews and focus groups, email correspondence, and posts from the web-based forum. Data analysis involved open, selective, and theoretical coding as well as the generation and subsequent analysis of memos. Coding was conducted manually using NVivo Plus (version 12; QSR International). Data collection and analysis continued until theoretical saturation was achieved and a theory had emerged describing a core concept, a number of related concepts, and how these concepts interact [42]. Study procedures were performed by MT and were in keeping with classic grounded theory as outlined by Glaser and Strauss [35], Glaser [42], and Holton and Walsh [43]. A summary of methods of rigor used, as outlined by Chiovitti and Piran [44], is presented in Multimedia Appendix 3 [44]. The Consolidated Criteria for Reporting Qualitative Research (COREQ) checklist [45] was used to guide the development of this report and can be found completed in Multimedia Appendix 4 [45].

### Ethics Approval and Informed Consent

Ethics board approval for this study was obtained through the Alberta Health Research Ethics Board (HREBA.CC-20-0429) before the initiation of study recruitment. Informed consent was obtained from all participants before study enrollment. The informed consent process included a discussion between potential participants and the researcher (MT) about the study objectives, methods, risks and benefits, and the option of study withdrawal at any point. These details were also outlined in the consent form. Participants were required to sign the consent form and return it to the researcher (MT) before study enrollment. All data collected were deidentified before analysis using a separate master list. Study data were only accessible to members of the research team. Participants did not receive compensation for taking part in the study.

## Results

### Participant and Study Activity Characteristics

Between August 2021 and June 2022, a total of 21 participants took part in 23 one-on-one interviews, 5 focus groups, and 26 web-based forum posts and sent the lead investigator a total of 10 emails responding directly to the study questions. In total, 38% (8/21) of the participants took part in a single interview or focus group, whereas 62% (13/21) participated in more than one interview or focus group. The average duration of the one-on-one interviews was 52 minutes and 30 seconds. The average duration of the focus groups was 57 minutes and 48 seconds. The demographic characteristics of the study participants are reported in Table 1.

**Table 1:** Participant demographics and cancer journey characteristics (N=21^a^).

Characteristics		Values
**Sex, n (%)**		
	Female	16 (76)
	Male	5 (24)
Age (years), mean (SD; range)		53 (15.3; 19-80)
**Marital status, n (%)**		
	Single	5 (24)
	Married	11 (52)
	Widowed	1 (5)
	Divorced	4 (19)
**Cancer type^b^, n (%)**		
	Breast	4 (19)
	Gynecological	4 (19)
	Hematological	4 (19)
	Lung	3 (14)
	Gastric	2 (10)
	Colon	2 (10)
	CNS^c^	2 (10)
	Prostate	1 (5)
	Sarcoma	1 (5)
	Thyroid	1 (5)
**Reported treatment intent^b^, n (%)**		
	Curative	11 (52)
	Noncurative	8 (38)
	Unsure	3 (14)
**Reported role, n (%)**		
	Patient	14 (67)
	Informal caregiver	6 (29)
	Both	1 (5)

^a^22 individuals consented to participate in the study, but 1 was unable to take part in any study activities because of reoccurring scheduling issues.

^b^Some participants reported multiple cancer experiences with more than one cancer type and treatment intent.

^c^CNS: central nervous system.

### Theory Summary

**Figure 1:**
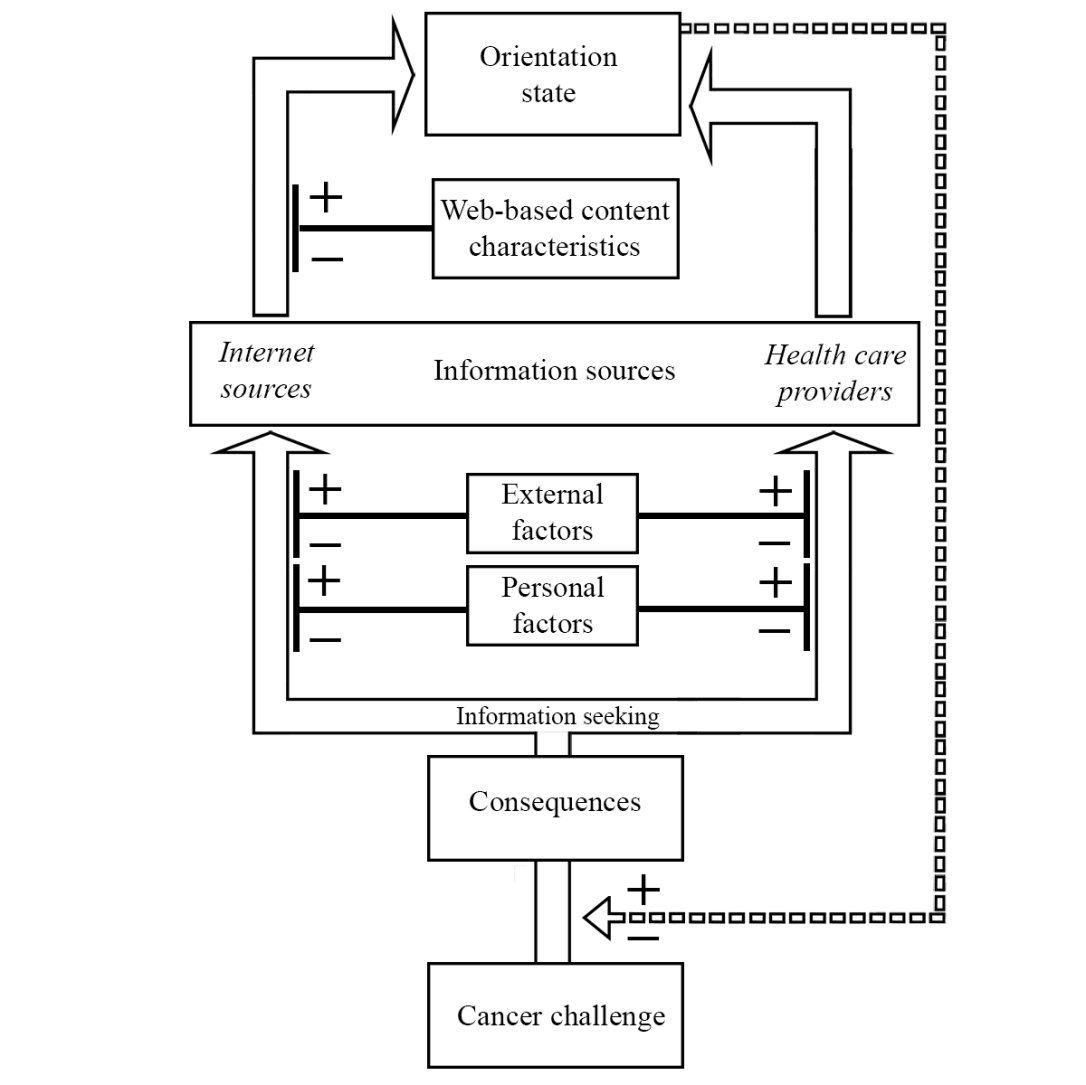
Model of orientation theory. Individuals seek information in response to facing challenges on the cancer journey. The internet and health care providers are both important information sources. Individuals may prefer to use the internet for information for a number of reasons including but not limited to ease of access, preference for anonymity, or lack of trust in their health care providers. The design of web-based content influences how well individuals are able to use it to become oriented. The state of orientation, in turn, influences the consequences of the cancer challenge being faced, including whether additional information seeking is necessary.

The theory that emerged consists of 6 interconnected concepts: (1) cancer challenges, (2) orientation, (3) cancer challenge consequences, (4) information sources, (5) personal and external factors, and (6) internet content design characteristics. Cancer challenges describe the challenges that individuals face resulting from a cancer diagnosis. Orientation, the core concept, describes the awareness individuals have of why a challenge is happening, what to expect, and the options that exist for dealing with the challenge. Cancer challenge consequences, or simply consequences, describe the impact that the cancer challenge has on an individual’s life and are ameliorated by how oriented the individual is to the challenge. Information sources are where individuals seek information from to become oriented, not limited to health care providers, television, the internet, family, and friends. Factors intrinsic to the individual (ie, personal factors) and external factors influence the individual’s preferences for accessing different information sources. Health care providers and the internet were identified as primary sources of information. Characteristics of accessed internet content affect how effective it is in helping the individual become oriented. The theory that emerged is subsequently referred to as orientation theory. A graphical model of orientation theory is presented in Figure 1. The following sections describe the properties and relationships of these concepts.

### Cancer Challenge

A cancer challenge describes the issues that are introduced into an individual’s life because of a malignancy. Participants described a wide range of challenges, ranging from planning travel to the cancer center and managing their day-to-day lives around the cancer diagnosis to dealing with uncertainty and existential issues regarding end of life:

...like how do we manage to get to these appointments and still maintain an income in the family and juggle all of these medical appointments and needs?

Participant 24

There were important questions about how quickly can you die from lung cancer...I think we became more acquainted in the death conversations as the journey became more clear.

Participant 5

Cancer challenges can be divided into 2 broad categories: direct and secondary. Direct challenges are related to the physical consequences of malignancy and participating in the receipt of health care. Examples include dealing with symptoms related to the malignancy, side effects of treatments, and navigating the health care system to get to appointments and treatments. Secondary challenges are those that arise as a result of direct challenges. They describe the collateral challenges of the malignancy diagnosis in the participants’ lives in their roles as employees, parents, spouses, and friends. The following 2 quotes illustrate examples of direct (ie, managing a cancer-related medical complication) and secondary (ie, maintaining a household income) challenges:

...the blood clot that I got in my leg which was actually the sign that we have to do some looking into what’s going on—so the blood clot came out of the blue and I [had] absolutely no idea that cancer and blood clots were related.

Participant 12

And I think that was probably one of the biggest challenges was managing on one income and I’m self-employed, so how do I work around getting to all of these appointments?

Participant 24

Participants described dealing with many cancer challenges throughout their cancer journey. Data analysis identified that certain cancer challenges more commonly occurred or became more prominent at typical times in the cancer journey. For instance, some of the common challenges participants described facing at the time of initial diagnosis included understanding why the cancer occurred, prognosis, figuring out which health care providers were going to be helpful, and planning their lives around the receipt of health care. Importantly, a common challenge participants experienced was finding information resources to help them navigate the cancer journey. This was especially true in the initial weeks to months between receiving the diagnosis and being connected with the cancer specialists who would be managing their care:

I [had] questions and so I didn’t have anyone, not my family doctor, not the specialist, not the surgeon, I didn’t have anyone that I could connect with and say: “hey I have questions.” “This is what I am concerned about.” “This is how I am feeling right now.”

Participant 10

Table 2 provides illustrative examples of the cancer challenges identified in this study, including their categorization as either direct or indirect and when they were observed to occur in the participants’ lives. Multimedia Appendix 5 contains an illustrative list of the cancer challenges identified through the coding and data analysis process but should not be considered a comprehensive list of cancer challenges that individuals experience.

**Table 2:** Categories, types, and timing of examples of cancer challenges.

Challenge categories and types		Observed timing^a^
**Direct**		
	Understanding what the diagnosis means	Beginning
	Making treatment decisions	Throughout active treatment
	Starting new treatments	Throughout active treatment
	Identifying which health care provider to see for which problem	Beginning and during active treatment
	Sharing diagnosis with friends and family	Beginning
	Managing new treatment side effects	Reoccurring with each new treatment
	Fear of reoccurrence	After active treatment
	Traveling to cancer care centers (eg, driving, parking, and lodging)	Beginning and active treatment
	Finding helpful sources of information to help navigate cancer challenges	Throughout the cancer journey
**Secondary**		
	Maintaining control of personal schedule	Diagnosis and active treatment
	Maintaining commitments outside of those related to receiving cancer care	Diagnosis and active treatment
	Performing in roles outside of being a patient with cancer or assisting a patient with cancer in receiving care	During active treatment and palliation

^a^Timing reflects general trends of when the challenge is common or most substantial; phases considered include beginning (includes diagnosis and initial treatment decision-making), active treatment (includes treatments that contain one or more types or multiple treatments in sequence), surveillance (occurs following active treatment with the intent of more anticancer treatment in the setting of reoccurrence or progression), and palliation (includes best supportive care and symptom management with no additional anti-malignancy–directed treatment).

### Orientation

Orientation describes the relative state of knowledge a person has regarding each individual challenge they face in their cancer journey. Broadly, the state of orientation an individual is in with respect to a given challenge can be described as oriented or unoriented. Individuals progress from a state of being unoriented to being oriented by developing the knowledge needed to address the questions they have regarding the cancer challenge. The questions participants described could be categorized into three groups of representative key orientation questions: (1) *Why is this happening?* (2) *What can I expect?* and (3) *What are my options for dealing with this?*

The first category of orientation questions relates to the nature of the challenge, including why something is happening or has happened. Examples include questions about why certain treatments are being recommended, why symptoms are occurring, and what has caused the cancer diagnosis. For instance, a participant described their experience of becoming oriented to why they developed lung cancer:

I [googled] why did I get it?...Like I am a non-smoker. I have never smoked in my life...Was I exposed to any of those chemicals [at work] and you know the answer was “no” I was just an office guy for all those years...I’ve got great genes—my mom died at 100 and my dad at 93...It’s just luck of the draw and I—I guess I was hoping for something a little more concrete.

Participant 12

The second category of questions (ie, *what can I expect?*) reflects the participants’ concerns about planning for the future and anticipating what kind of challenges they will face. Examples include questions about life expectancy, what the side effects of treatments will be, and the timing of appointments. For instance, a participant who was an informal caregiver of older parents who had passed away shared the following:

I remember needing to find out the prognosis...especially for my dad, [for my] mom it was just three weeks [after the diagnosis] and she was dead. For my dad it was five years and so for him, periodically, I would check in like, has the research changed?

Participant 5

The third category (ie, *what are my options?*) reflects the participants’ search for answers regarding what can be done to optimize or improve the outcome of the cancer challenge they are facing. Participants described looking for answers regarding treatment options (including conventional biomedical treatments and alternative and complementary options), exercise, nutrition, and other strategies to manage the many different types of challenges. For instance, a participant described searching for options to minimize chemotherapy toxicity:

I did ask [in the information session], I said “is there anything else I could do or any supplements I can take that would help to boost or build my immune system?” because there is no mention of it whatsoever in any of the [information that was provided].

Participant 10

### Consequences

The relative state of orientation has important consequences for the well-being of the individual facing the challenge. Participants described more negative consequences of managing cancer challenges when in an unoriented state compared with an oriented state. An unoriented state was associated with increased uncertainty about what to expect and what action to take and a negative impact on the time, emotional and physical energy, and financial resources they had available to use for other aspects of their lives. For instance, a participant described the impact of being unoriented regarding how to manage a common side effect of chemotherapy and how information from a health care provider helped them become oriented and avoid the problem in the future:

I had a problem after the IV infusion that I would feel like my throat had closed off and I could not breathe—very scary as I thought I was going to pass out. I could not find anything about this side effect. My oncologist was able to tell me some of his understanding of the side effect and how to avoid future problems. [Participant 21]

Another participant, an informal caregiver, shared their experience working with a health care provider to manage their partner’s seizure medications and how becoming better oriented to the limitations of the health care provider’s scope of practice and the resources available improved their cancer experience:

[The specific health care provider] only prescribes and knows a couple of very common [seizure medications]...because the first medication...wasn’t working, [they] added a benzodiazepine, and then just kept on upping it, and so [the patient] was having pretty detrimental side effects from the benzos entirely, and they also weren’t working for seizure control...[it] took months [to find a neurologist]...the next time that we needed adjustments to medication...we just straight up, just went back [to the specific health care provider] and said, “We wanna see [the neurologist].”

Participant 9

Importantly, just as cancer challenges were identified as occurring concurrently throughout the cancer journey, the consequences of cancer challenges did not occur in isolation. Participants described being overwhelmed, experiencing intense emotions, and being essentially unable to function at times when they faced many challenges at once—especially if they were unoriented to several of the challenges they were facing. A participant, an informal caregiver, shared their experience following the diagnosis of their partner:

Yeah, honestly, I think at the time, I don’t know if I was feeling much for emotion [I] was just totally overwhelmed. We went from a diagnosis of, “Yes, we believe this is lymphoma,” [to] starting chemo because it was stage four [two weeks later]. So it was very fast and very overwhelming and...Yeah, I mean the dread, the fear, the unknown, it was really so challenging...The financial aspect was terrifying, what are we gonna do? My partner had no health insurance and no backup savings or anything like that, and so that was really challenging. And yeah, so feeling very helpless, very alone in trying to navigate things.

Participant 24

Textbox 1 provides a summary of the consequences described by participants as they faced cancer challenges from different states of relative orientation.

**Consequences of managing cancer challenges from different orientation states.**

**Unoriented**

- Being unsure of how to act and increased chances of making a regrettable choice
- Not knowing what to expect
- Not being able to provide others with accurate information
- Increased fear, anxiety, stress, and pessimism
- Increased requirements of time, energy, and money to deal with challenge
- Less effective in participating in management of health issues

**Oriented**

- Awareness of right choice of action
- Knowing what to expect
- Able to help orient others
- Reassurance and hope
- More efficient use of personal resources, including for finding information
- More effective role in managing health issues

### Information Sources

Obtaining the information needed to become oriented to a cancer challenge occurs in many ways. Participants described receiving information from multiple sources, including friends and family, television, and books or audiobooks. Personal experience was also an important source of information, particularly for addressing the key orientation question of *what can I expect?* A participant shared their experience with treatment and how they came to learn that their reaction to treatment was unique:

...so you know the treatment has not really affected me I see people come in and they are very [emaciated] and they have no hair and they are very sluggish...[I find] that half way [through treatment] I have a nap, at the first of the treatment and then I’m like ready to tear apart the place I am just so full of energy...it’s been just the opposite for me I guess than it has been for a lot of people.

Participant 13

Of all the potential sources of information, participants consistently identified health care providers as an important source of information. With few exceptions, participants described that they trusted the information that health care professionals provided the most compared with other sources. However, the internet was also consistently described by participants as an equally essential source of information.

### Personal and External Factors Influencing Information Source

Participants described several factors influencing their choice to use internet content for information as opposed to health care providers. These can be divided into the categories of personal and external factors. Preference for exploring content related to cancer challenges anonymously, respect for the health care providers’ time, or being in an overwhelmed state at the time of the health care provider visit were some of the personal factors described. A participant described their experience obtaining information from their health care provider:

It wasn’t Pollyanna because at that time [of the oncologist visit] you are absolutely on overload already...I wouldn’t have found [more information] useful because you are already up to your shoulders and you just keeping your head above water to help you exist.

Participant 3

External factors such as the characteristics of the health care system (ie, clinic location and operational hours) as well as the attitudes and language used by health care providers were important in determining the participants’ choice to use the internet as a potential source. Among these factors, accessibility of health care providers in terms of geographic location, appointment availability and duration, and general convenience were commonly identified as factors that influenced internet use:

Yah, basically I think as a patient, if I could like email my [health care team] I think there would be a lot less random googling, you know?

Participant 15

Importantly, internet content was not only accessed when participants were unable to use information from health care providers because of personal or external factors. Even when health care providers had given participants potentially useful information for helping them address a cancer challenge, the internet still played an important role for many in becoming oriented. A common practice described by the participants was to use the Google search engine to verify the information they received from health care providers, non–health care providers, and elsewhere on the internet. This practice involved looking for additional sources to compare whether the information was consistent. Participants described that, when the information was consistent between sources, they considered the information accurate and the sources credible. In contrast, inconsistent patterns raised questions of doubt. For instance, an informal caregiver described their experience with a health care provider whom they ultimately determined was not credible:

...I was looking for other sources of information to see if I could validate or discredit what [the oncologist] was telling [the patient]. And then when I found things online then I went to my [family physician] and asked more questions because that was someone that I trusted, and I didn’t trust [the patient’s oncologist] and it’s a good thing we didn’t.

Participant 2

Situations where internet searching was preferred to obtaining information from health care providers could be divided into 6 categories, summarized in Table 3.

**Table 3:** The 6 situations where participants preferred web-based information sources to health care providers.

Categories	Descriptions	Supporting participant quotes
Accessing routine health services	Looking up things to assist with accessing health care services (ie, directions, phone numbers, hours of operation, parking, and lodging)	“Sometimes it just basic as getting peoples’ phone numbers, so, I might have commented already on that in the blog. But you cannot find palliative care’s phone number online anywhere.” [Participant 5]
Accessing additional services outside of what the health care team routinely provides	Looking up how to access health care services not provided through consultation or referral from a health care provider in the public health care system (eg, massage therapy, self-referral physiotherapy, naturopathy, and medical assistance in dying)	“Why else did I go to the internet? Sometimes just practical stuff like for a lot of the homecare needs, you know where do you find, you know, a wheelchair and how does that process work? Just the practical details of all of the associated equipment and supplies that were needed because that is not in one place, and it is hard to find.” [Participant 5]
Cannot access health care providers	Addressing questions that arise in between or after appointments	“You know you leave the oncologist’s office and it’s like ‘oh shit I should have asked [them] about this’ and so I go home and do that kind of searching.” [Participant 12]
Questions on which a health care provider likely will not be helpful or may be hurtful or where there is a preference for anonymity	Questions are out of the provider’s scope or not relevant to the specific clinical interaction, or responses are not expected to be helpful.	“I had an issue with the eating...as a big [person], I am programmed not to eat stuff different things...I wouldn’t talk about it anymore with the doctor for sure...they are going to be like ‘what is wrong with you?’ you know it is just going to make me feel bad and life is too short now.” [Participant 10]
Validate or fact-check information from health care providers or other sources	These questions are related to confirming information received from health care providers, other individuals, or other sources—such as the internet.	“...so trying to sort out and match what was being told to us by physicians with what the literature was saying out there and seeing if it matched. So a little bit of triangulating, like trying to figure out you know what my parents were saying, what the doctors were saying when I was able to sit in on appointments with either of them and then what I was able to read on the internet.” [Participant 5]
Questions not directly related to the care of the individual living with cancer or the care of a loved one	These questions might include those related to opportunities for public advocacy or improving cancer care for the future.	“I am also part of a support group here in Calgary for Lung Cancer patients, and there are triggers that could come out of that. Somebody will say something about ‘oh there is this new brigatinib drug’ which is like the next level up for me, ‘oh maybe I should look that up.’” [Participant 14]

### Internet Use Patterns

#### Internet Use Timing

In contrast to information accessed through health care providers, internet resources are generally accessible around the clock and without travel. Internet information gathering commonly occurs in between other activities that either cannot be rescheduled or are of higher priority. However, participants also described rearranging their schedules and setting aside time to facilitate web-based information gathering to address orientation questions that they considered to be high priority. As an example, an informal caregiver described transitioning from searching in between other tasks while “on break” (participant 5) at work to scheduling time to sit down to find specific information. This occurred when the individual was struggling to address the key orientation questions of *what to expect?* and *what are my options for dealing with this?* after having a disappointing experience with health care locally:

So that became a lot more specific in terms of setting aside half an hour to sit down and figure out “who am I going to call at this [out of country] clinic? What information do they need before I call? What do I need to have next to me?”

Participant 9

#### Sources and Strategies for Finding Web-Based Information

In general, participants identified that internet information gathering included using search engines, browsing familiar sites, scrolling through social media feeds and discussion boards, and accessing web-based patient portals. Participants described different sources as being useful for identifying different types of information. Social media sources were helpful for connecting with people who had experienced similar cancer journeys, especially in the setting of rare malignancy types, for peer support, including first-person accounts of what to expect and direction to helpful resources:

So Facebook I find to be helpful, Twitter, Instagram, TikTok. TikTok is the one when I go on and talk about what I live with and stuff and I blog as well and I do YouTube.

Participant 19

However, some participants felt that social media and discussion forum content were untrustworthy and avoided them. As a participant stated, “...not TikTok or whatever. I refuse to believe anything that’s on there” (participant 14).

A few participants identified that recommendations for internet sites were provided directly by health care providers or indirectly through pamphlets and handouts provided through health care system facilities. However, Google searching was identified consistently by participants as the primary approach for finding web-based information. Participants described using the Google search engine to conduct searches using several keywords related to the cancer challenges they were facing and then browsing search results and selecting those that were assumed to be helpful based on previous experiences with the site, familiarity with the website domain name, or previous recommendations from health care providers.

### Web-Based Cancer Content Design: Challenges Experienced With Web Page Content

#### Rabbit Holing

Participants described that, when they began searching for information about a cancer challenge, they would come across unfamiliar terms and concepts. They would then redirect their internet searches to further explore these new concepts. This process involved clicking on links discovered on websites or conducting new searches related to the unfamiliar terms. Inevitably, they would end up not addressing the information need related to the initial search. The process, described by the participants as “rabbit holing,” was eventually terminated when the individual was interrupted by another task or became emotionally exhausted. Going down the rabbit hole was identified as a distracting and undesirable event. A participant described their experience as follows:

...you get in that rabbit hole, you click...And then you click, and you click, and you click, and you click, and I’ve done that before myself. And all of a sudden I’m like, “Oh, I actually came here to look up whatever, and an hour and a half later, I’m on some other random site that I’ve just gone down this rabbit hole.”

Participant 24

#### Lack of End User–Oriented Design

On multiple occasions, participants described accessing web-based content intended to provide a comprehensive overview of a topic but finding the content presented in a way that was problematic. Common issues were too much content, nonintuitive organization and layout, or lack of details specific enough to help the individuals address the key orientation questions. A participant described their experience with a website from a prominent Canadian health center:

...you go looking for a certain type of information it does not bring you to what the next logical step is. It is like you have to go really deep into the [website] to find the one piece of the information you are looking for and it shouldn’t be like that.

Participant 9

Participants also described that, without warning, they came across information that was distressing or that they were actively trying to avoid, such as information on prognosis. In addition, content irrelevant to the cancer journey of the individual was often presented on websites from well-regarded cancer centers, including targeted advertisements on web pages designed for people living with cancer. This was identified as a source of distraction that was upsetting to some participants.

## Discussion

### Principal Findings

The cancer journey presents patients and informal caregivers with many new and unfamiliar challenges. The challenges are numerous and varied and include those directly related to engaging with the health care system as patients and informal caregivers and those related to navigating roles as parents, spouses, friends, and employees outside the cancer context [25]. How well an individual is oriented to these challenges while navigating them has important consequences for the individual’s well-being and overall cancer experience [10,16-18]. Although health care providers are an important source of information, the internet may be a preferred source depending on the challenge the individual is working to become oriented to, as well as the characteristics of the individual, their health care providers, and health care system.

The characteristics of web-based content affect how useful it is for helping individuals become oriented to the cancer challenges they face. The presence of distracting links, unfamiliar terms, and distressing content; the lack of intuitive design; and the absence of information addressing all or any of the key orientation questions are characteristics expected to make web-based content less useful. On the basis of the findings of this study, five recommendations for creating web-based content that supports orientation are as follows: (1) clearly identify the cancer challenge and population the content is addressing as well as the presence of any potentially distressing information; (2) provide versions of the content in different formats (eg, printer-friendly, audio, video, and alternative languages); (3) state who created the content, including the individuals, organizations, and processes involved; (4) place hyperlinks after the 3 key orientation questions have been systematically addressed; and (5) ensure that content is optimized for discovery by search engines, especially Google.

An infographic outlining these recommendations can be found in Multimedia Appendix 6. A detailed discussion of how these recommendations were informed by orientation theory is included in Multimedia Appendix 7 [46-54]. Multimedia Appendix 8 [55,56] includes sample web-based content developed through the course of the study with the participants, along with an explanation of how it reflects the principles of orientation theory and the 5 recommendations for web-based content design.

### Building on Existing Theories

Orientation theory is a substantive middle-range theory addressing information-seeking behavior in the cancer context with implications for guiding web-based content design [1] that complements existing theoretical work, including that of Wilson [21] and Longo [38]. Both Wilson [21] and Longo [38] connect information-seeking behavior and information needs with important consequences. Wilson [21] describes that information seeking and information behavior in general are an important part of effectively dealing with stresses. Longo [38] links addressed information needs with themes of empowerment, satisfaction, increased participation in activities of daily living, and improved health outcomes. Similarly, the consequences of orientation (Textbox 1) include empowerment through the ability to participate actively in care (including self-management); enhanced emotional well-being; and improved participation in the roles and relationships existing outside of health care receipt, such as those with friends, family, and the workplace.

Both Wilson [21] and Longo [38] identify that individuals obtain information from a number of different sources, but neither of these theories detail why individuals living with cancer use the internet. Orientation theory adds to these works by both identifying the importance of information from health care providers and characterizing the internet as a uniquely important source of information in the cancer context that is preferred in some instances (Table 3). In addition, orientation theory highlights the important process that individuals engage in to validate information by cross-checking the information they receive from sources, including health care providers, with content on the internet. These findings underscore that internet-sourced content is not just complementary but is an essential source of information for many individuals living with cancer.

### Clinical Implications

Orientation theory describes health care providers as both a source of information and an influencing factor on information source preferences. This places clinicians in a position to both provide information and influence which sources are accessed by individuals. Therefore, clinicians should consider providing direction to useful, credible websites and facilitating access to specialized staff such as nurse educators as part of routine practice. In addition, the provision of educational content that assists patients and informal caregivers in becoming better skilled at evaluating the quality of web-based content may be a welcome addition for many individuals experiencing cancer as this will likely go a long way toward helping them navigate the many challenges not brought to the attention of their care providers. In particular, web-based resources that individuals living with cancer may find helpful include *Health On the Net* [57], which provides a search engine restricted to certified high-quality web-based health information, and *DISCERN* [58], which provides a tool and instructions developed to help health care consumers evaluate the quality of written health information.

Finally, orientation theory states that individuals may have varying levels of comfort with health care providers and prefer to explore some topics anonymously or outside the clinic. As a result, clinicians should not assume that just because a patient or informal caregiver does not ask about a topic, they do not have unanswered questions about it. Orientation theory suggests that clinicians should consider voluntarily providing information, including written material or direction to web content, that can be reviewed outside the clinical setting. This is especially true for cancer challenge topics of a sensitive nature that may have a major impact on both the patient and informal caregiver, such as end of life [59] and the impact of cancer and cancer treatment on sexuality [60].

### Research Implications and Future Directions

Identifying that orientation has multiple consequences and involves finding answers to multiple questions raises concerns about appropriate study measures for evaluating the effectiveness of informational interventions. A scoping review examining existing validated information needs assessment tools developed in the cancer context will hopefully provide some insight into which questionnaires best reflect the concepts outlined by orientation theory [61]. However, additional work is needed to explore how the identified consequences of orientation are reflected in existing instruments.

On a larger scale, how to address information needs in a way that results in a meaningful improvement in the cancer experience remains an important question. This study provides an important theoretical starting point [33] by describing the concept of “cancer challenges” and the process and consequences of orientation. However, it does not attempt to provide an exhaustive list of the cancer challenges that an individual is likely to face in their cancer journey. This study identified that cancer challenges occur concurrently and that they may be able to be grouped by their stereotypical temporal relationships. Therefore, it is likely that the most impactful interventions will be designed to support orientation to multiple cancer challenges at once. To accomplish this, research is needed to systematically map out the cancer challenges that individuals face in their journey, including when they are likely to arise, to inform subsequent intervention development.

Finally, an important consideration relevant to both coping with cancer and information-seeking behavior is the distinction between high and low monitors (ie, blunters) [62]. The literature supports that individuals can be dichotomized into 1 of these 2 coping styles, with each having important implications for how an individual navigates health concerns. High monitors have been characterized by being more likely to seek out information about their illness, whereas low monitors typically avoid seeking information [62]. There is some evidence suggesting that these coping styles may be, at least in part, situational [63], with individuals exhibiting blunting behavior in response to some stressors and high monitoring behavior in response to others. Given the considerable number of cancer challenges that the participants in this study identified, it is certainly possible that there are specific challenges that an individual may preferentially seek out information for at any given time while ignoring others. However, this was not explored in any detail in this study. Exploring the relationship between cancer challenges and coping styles in future research is important as it may have implications for both evolving orientation theory and informing how to best develop and deliver informational interventions.

### Limitations

Glaser and Strauss [35] identify that theory produced using the classic grounded theory approach is robust and valid as it emerges from data obtained directly from the field of interest. However, there are a number of important considerations in terms of interpreting and applying the findings of this study. First, the data used in this study were collected from participants who had internet access in a geographic region where health care is administered through 1 body (ie, Alberta Health Services). In addition, strategies guiding participant selection for data collection were driven primarily by age, role as either patient or informal caregiver, cancer type, and curative versus noncurative intent. The role of factors such as ethnicity, sex, and gender was not explored. Given the similarities between orientation theory and other preexisting theoretical work [20,21,38,40], it is likely that the identified concepts and their relationships are relevant across a wide range of populations. However, the concepts described in this paper, such as the consequences of orientation, likely manifest differently in different contexts. Therefore, some caution should be exercised when applying the concepts of orientation theory to develop content or guide other interventions as the concepts may not be universally applicable. For this reason, including individuals from the target audience in content or intervention development is likely key to ensuring that the content is both applicable and appropriate [64].

Finally, internet use in orientation theory was primarily focused on web page content. This was because web pages discovered through Google searches were identified as the primary source of web-based content for the participants, with other sources playing a lesser and more inconsistent role. As a result, these other sources of internet content were not explored after the conclusion of open coding [35]. Therefore, although it is certainly possible that the insights gained in this study are relevant across other media, such as social media, patient portals, discussion boards, and paper-based content, content creators should exercise caution when applying them outside web page design.

### Conclusions

Through the lens of orientation theory, the cancer journey can be viewed as one that involves navigating many unfamiliar and often unwanted challenges, often simultaneously. How informed individuals are of why each challenge is occurring, what to expect, and the options for managing it has important implications for the individual’s well-being and cancer experience [5,10,18,65]. The high prevalence of unmet information needs of both patients and informal caregivers suggests that there is considerable opportunity for transforming the cancer experience by improving information provision [8-10]. The internet has the potential to be a source of low-cost, high-quality, and easily accessible information capable of improving the journey of many individuals living with cancer. However, to create robust and effective web-based informational interventions, further work is needed to fully understand the cancer journey, the many challenges faced, and how to assess the consequences of orientation. In the meantime, cancer clinicians and creators of web-based cancer content must recognize the power of information to transform the cancer journey and their responsibility to share information in a way that does no harm.

## Appendix

Multimedia Appendix 1 Intake survey.

Multimedia Appendix 2 Initial interview guide.

Multimedia Appendix 3 Rigor statement.

Multimedia Appendix 4 Completed Consolidated Criteria for Reporting Qualitative Research (COREQ) checklist.

Multimedia Appendix 5 Cancer challenge example list.

Multimedia Appendix 6 Infographic for better web-based content.

Multimedia Appendix 7 Theory-based discussion of the 5 recommendations for better web-based content.

Multimedia Appendix 8 Theory-based explanation of generated sample content.

## Abbreviations

COREQ: Consolidated Criteria for Reporting Qualitative Research

## Data Availability

The data sets generated during this study are not publicly available as they contain sensitive information that may make it possible to identify study participants despite careful steps being taken to remove identifying details. Data may be made available upon reasonable request from the corresponding author with ethics board consultation.
