# Supplementary material for: Navigating the Cancer Journey Using Web-Based Information: Grounded Theory Emerging From the Lived Experience of Cancer Patients and Informal Caregivers With Implications for Web-Based Content Design
Source: JMIR Cancer. 2023 May 17;9:e41740. doi: 10.2196/41740 (PMC10233434; doi:10.2196/41740)
Supplement: Multimedia Appendix 1 [file cancer_v9i1e41740_app1.docx]

Multimedia Appendix A – Intake Questionnaire

# Intake Questionnaire

1. Please complete this survey at the time of completing your consent form. Its purpose is not to exclude individuals, but to ensure a diverse group of people are included in this study.
2. This questionnaire uses the term “living with cancer” to refer to individuals that have either received a diagnosis of cancer, supporting someone that is, or have been affected by someone else cancer diagnosis.

Year of Birth: Gender:

Postal Code:

Marital Status:

| Single | Married/Common Law | Divorced | Widowed |
| --- | --- | --- | --- |

Education Level:

| Some High School | Graduated High School | Some University/College | Graduated University/College |
| --- | --- | --- | --- |

Is English your first language?

| YES | NO |
| --- | --- |

Were you born in Canada?

| YES | NO |
| --- | --- |

If you were not born in Canada, how many years have you lived in Canada:

|  |
| --- |

Annual Household Income:

| Less than $20,000 | $20,000 to $50,000 | $50,000 to $100,000 | More than $100,000 |
| --- | --- | --- | --- |

You are completing this questionnaire as a:

| A cancer patient | Friend of a cancer patient | Family of a cancer patient |
| --- | --- | --- |

Is this your first experience with cancer?

| YES | NO |
| --- | --- |

Do you have thoughts or ideas about how you could be better supported as someone living with cancer either as a patient, or supporting a patient?

| YES | NO |
| --- | --- |

Do you consider yourself to be “computer savvy”?

| YES | NO |
| --- | --- |

Do you use email regularly?

| YES | NO |
| --- | --- |

Do you use the internet regularly for web searching?

| YES | NO |
| --- | --- |

Are you comfortable participating in small group discussions?

| YES | NO |
| --- | --- |

Would you be comfortable participating in an online videoconference (equipment will be provided if you do not already have access to the internet or web-conferencing equipment)?

| YES | NO |
| --- | --- |

Would you be comfortable providing information through email correspondence?

| YES | NO |
| --- | --- |

What kind of cancer do you, or the person you are supporting have? (please fill in the blank)

|  |
| --- |

Is the cancer being treated with curative or non-curative intent?

| Curative | Non-curative | Unsure |
| --- | --- | --- |

| For Researcher Use Only  Study Number: |
| --- |
