# Supplementary material for: Navigating the Cancer Journey Using Web-Based Information: Grounded Theory Emerging From the Lived Experience of Cancer Patients and Informal Caregivers With Implications for Web-Based Content Design
Source: JMIR Cancer. 2023 May 17;9:e41740. doi: 10.2196/41740 (PMC10233434; doi:10.2196/41740)
Supplement: Multimedia Appendix 2 [file cancer_v9i1e41740_app2.docx]

Multimedia Appendix 2 – Interview Guide

# Guiding Questions for Focus Group #1

Estimated time: 105 minutes (15 minute introduction, 45 minute sessions, 15 minute nature/refreshment break, 45 minute session)

Introduction – outline background to the project, what the purpose of this project is.

Part 1 Questions (45 minutes):

1. Let us go around the room, please share your name, where you are from, and why you agreed to participate in this study?
2. When was the very first time you remember being confused or uncertain about something related to cancer? (follow-up questions: Why was that issue important to you? Were you able to find clarity? How did you find it?)
3. What kind of challenges does cancer present to your life?

Break – 15 minutes

Part 2 Questions (45 minutes):

1. People find different types of information helpful for planning their lives. What kind of information have you used to plan your life around receiving support or supporting someone with cancer? What was helpful, what was not?
2. We will now look at some specific examples of informational content that someone with cancer might access or be provided with. Can you tell me how these examples would be helpful? Can you tell me what could be changed to make this content more helpful to help you plan your life? – Show examples of different cancer specific content including websites, handouts, email newsletters, social media posts.
3. In seeking to understand how different types of information are useful for planning one’s life following cancer, is there anything that you would like to share?
4. Let’s go around the room, and take turns summarizing todays discussion…
5. Have we missed anything? Is there anything that I should have talked about but didn’t?
6. Is there anything that regarding to informational resources related to living to cancer that we didn’t’ talk about that you feel is important for cancer patients?

At the conclusion of the focus group, participants will be encouraged to communicate any additional thoughts and share examples of helpful resources with the researcher electronically in the next seven days.
