# Supplementary material for: Navigating the Cancer Journey Using Web-Based Information: Grounded Theory Emerging From the Lived Experience of Cancer Patients and Informal Caregivers With Implications for Web-Based Content Design
Source: JMIR Cancer. 2023 May 17;9:e41740. doi: 10.2196/41740 (PMC10233434; doi:10.2196/41740)
Supplement: Multimedia Appendix 3 [file cancer_v9i1e41740_app3.docx]

Multimedia Appendix 3 – Rigor Statement

| Implementation of eight methods of research practice for enhancing rigor in grounded theory research. Adapted from Chiovitti et al. 2003^†^ | | |
| --- | --- | --- |
|  | **Suggested Method** | **Implementation Approach** |
| **Credibility** | Let participants guide the inquiry process | Participants were asked primarily open ended questions. The study direction, including the concepts that were explored, evolved based on data analysis of the open-ended questions and what participants expressed as being important. |
|  | Check the theoretical construction generated against participants meanings of the phenomenon. | Throughout the study the emerging concepts were reviewed explicitly with the study participants in one-on-one interviews, focus groups, and online forum discussion. The emerging concepts were used to guide the development of sample online content with participants, and the feedback received from the initial drafts was used to revise both the content and inform the emerging theory. |
|  | Use participants' actual words in the theory | Short and long quotes from participants are used throughout the presentation of the theory. Several concepts were either named using participants words (i.e., rabbit holing) or terms that were confirmed as helpful by participants (i.e., validation). |
|  | Articulate the researcher's personal views and insights about the phenomenon explored. | The researcher (MT) is a practicing medical oncologist who has a research interest in understanding how to better support the information needs of those living with cancer. This research project was conducted as part of PhD thesis work. He has conducted three previous classic grounded theory studies, exploring how identify is impacted by the cancer diagnosis, what makes quality information, and the variables the define and impact the cancer experience. He views the information that is available to patients and their friends and families through the lens of empowerment/oppression in keeping with the concepts of critical education theory outlined by Paulo Freire. |
| **Audibility** | Specify the criteria built into the researcher's thinking | The analysis of collected data was done within a structural symbolic interactionist framework informed by identity theory. Coding and memoing took into account how social structure, and the roles and relationships that the individual’s maintained were impacted by the cancer journey, and participation with the healthcare system. |
|  | Specify how and why participants in the study were selected | The recruitment approach was designed to include individuals with a varied cancer experience. Participants were selected for specific study activities based on their demographic and cancer characteristics to ensure that concepts were explored in various contexts. This process involved inviting specific individuals for focus groups and one-on-one interviews based on the responses given on the intake questionnaire. |
| **Fittingness** | Delineate the scope of the research in terms of the sample, setting and the level of the theory generated | Please see Table 3 for sample characteristics. The theory developed is a middle range theory. |
|  | Describe how the literature relates to each category which emerged in the theory. | How the main concepts of this theory add to the existing literature are outlined in the discussion section. |

^†^Chiovitti RF, Piran N. Rigour and grounded theory research. Journal of Advanced Nursing. 2003;44(4):427-35. doi: <https://doi.org/10.1046/j.0309-2402.2003.02822.x>.
