# Supplementary material for: Navigating the Cancer Journey Using Web-Based Information: Grounded Theory Emerging From the Lived Experience of Cancer Patients and Informal Caregivers With Implications for Web-Based Content Design
Source: JMIR Cancer. 2023 May 17;9:e41740. doi: 10.2196/41740 (PMC10233434; doi:10.2196/41740)
Supplement: Multimedia Appendix 5 [file cancer_v9i1e41740_app5.docx]

Multimedia Appendix 5 - Cancer Challenges Identified Through Data Analysis

As noted in the manuscript, this list is not exhaustive of all of the challenges related to the cancer journey that individuals experience. It is provided to further illustrate the concept of cancer challenges and was generated from the relevant open codes resulting from data analysis process.

**Direct Challenges**

Working with healthcare providers

Understanding who to ask for what

Navigating interactions with unfamiliar providers

Finding information to assist with

Preparing for appointments

Getting needs met despite limited time with providers

Finding strategies for effective information sharing

Trusting healthcare providers

Understanding prognosis

Treatment

Understanding the intent of treatment

Understanding steps in treatment

Understanding the side-effects/toxicities of treatment

Understanding how response is assessed

Is this the right treatment for me/patient?

What are the treatment options?

How will I/the patient be cared for during treatment?

What do I need to do during treatment?

Transitioning between phases of the caner journey

Managing cancer symptoms

What does this new symptom mean?

Surveillance

What are the signs of re-occurrence

Minimizing the chances of re-occurrence

What should foods to eat versus avoid

What supplements can be taken

What do the test results mean

Navigating (new) healthcare systems

Where can I find additional supports outside of what my healthcare providers are offering

Getting to and from medical care?

Parking

Lodging

Travel

Managing emotional well-being

Dealing with being overwhelmed

Living with uncertainty

Fear

Finding and using information

Is this information trustworthy?

Where can I find reliable information?

**Secondary Challenges**

Coordinating life goals around healthcare provider appointments and treatments

Managing unsolicited/unwanted opinions regarding cancer treatment from friends, family, and strangers

Sharing diagnosis with friends, family, employers

Dealing with employers

Dealing with finances

Minimizing the negative impact of the diagnosis on relationships

Maintaining a household

Helping others cancer survivors
