# Supplementary material for: Navigating the Cancer Journey Using Web-Based Information: Grounded Theory Emerging From the Lived Experience of Cancer Patients and Informal Caregivers With Implications for Web-Based Content Design
Source: JMIR Cancer. 2023 May 17;9:e41740. doi: 10.2196/41740 (PMC10233434; doi:10.2196/41740)
Supplement: Multimedia Appendix 7 [file cancer_v9i1e41740_app7.docx]

# Five Recommendations for Better Online Cancer Content

Orientation theory identifies that the internet plays an important role in helping individuals living with cancer understand the many challenges they face, including what options they have for managing them. Additionally, it identifies that the characteristics of internet content are an important determinant of whether the content is helpful in assist individuals in orienting to a specific cancer challenge (see Figure 1 of the accompanying manuscript). To date, specific guidance for how to optimally design online content to assist individuals on the cancer journey do not appear to have been developed. The following five recommendations are informed by orientation theory and are expected to minimize some of the challenges individuals experience with existing online cancer content.

## Clearly identify the specific cancer challenge being addressed, who the content is for, the presence of any potentially sensitive information, and systematically address the key orientation questions.

The cancer challenge and target audience the content is designed for should be clearly identified so the end-user can easily determine if the content is applicable to them and whether they want to review the content. The presence of potentially sensitive information (e.g., content related to prognosis) should be highlighted to provide caution to the individual and help them decide if they want to navigate through the content, come back to it at a later point, or avoid it all together. This recommendation reflects findings from this study and others [46-48] that online health content is not benign and may be a potential source of harm. Without clear clues about the nature of the content and who it is for, individuals may not be able to discern whether information is relevant to them resulting in an unnecessary time used to consume the content or misdirection in terms of the actions they take to navigate the cancer journey.

Additionally, each piece of content should be designed to address the three key orientation questions (i.e., *why is this happening*, *what can I expect*, and *what are my options*), as they pertain to the cancer challenge being addressed. This approach supports individuals in becoming oriented by systematically addressing the key informational aspects of each challenge necessary for becoming oriented. In additional, this approach provides a consistent framework to help individuals more easily navigate the content itself and identify gaps in the content being presented.

## Content to address a specific cancer challenge should be developed in multiple languages, literacy levels, and audio/visual formats.

Content creators should endeavour to present content in language and formats that are easily understandable and accessible to the target audience. This recommendation reflects the concept outlined by orientation theory of personal factors being a potential barrier to accessing informational sources, including those on the internet. In addition to meeting increasingly recognized online accessibility standards [49, 50], non-English translations, and printer friendly versions should be made available for content whenever possible. Participants in this study identified that the web-based content they found was consumed in a number of ways and was also accessed to provide information to other individuals – such as in the case of adult children providing printed information to elderly parents. Therefore, web-page content addressing a specific cancer challenge should also be created in multiple alternative formats linked to the webpage to overcome accessibility barriers including those presented by language, literacy, and learning styles.

## The processes, organizations and individuals involved in creating the content should be clearly identified.

Content should clearly identify both the organization and authors responsible for creating it, including relevant credentials and affiliations. This is important because individuals in the orientation theory study identified that, in addition to comparing informational content from multiple sources, they also considered the credibility of the source in terms of weighing the accuracy of the content. This recommendation also aligns with published recommendations for evaluating online health information quality [51]. Additionally, the content should clearly identify how it was developed. In particular, the roles and contributions of patient/informal caregivers and healthcare professionals should be outlined. This is important, as those with lived experience will be oriented to the cancer challenge and have different perspective to share then healthcare professionals. These practices in reporting should not be seen as barriers to content creation for those without professional credentials, or organizational affiliations. Instead, they should be considered necessary steps to ensure transparency and assist individuals consuming content in fully assessing how to interpret the content and apply it to their own journey.

## Hyperlinks should be placed at the end of content and should include links to both other sources addressing the same cancer challenge as well as related cancer challenges.

Including hyperlinks to help end-users identify similar and related content elsewhere on the internet is an important part in creating useful online content. Links to content from other sources addressing the same cancer challenge is a key step to helping end-users verify what is accurate, by supporting them in comparing information across multiple sources. Similarly, including links to other cancer challenges that the individual is likely to be facing at the same time (e.g., early on the cancer journey versus when transitioning to surveillance versus when transition to palliative care) will assist individuals in becoming oriented to the other cancer challenges they will be likely to face. The importance of this is described by orientation theory, as it was found that the participants frequently expressed navigating multiple challenges at once, and these challenges could be predictably grouped.

However, an important challenge that the participants faced was that hyperlinks were embedded in online content in a way that was distracting and lead them away from working through the content that was being presented (i.e., “rabbit holing”). To address this, hyperlinks should be placed outside of the body of the content being presented, after the key orientation questions have been address. This approach to content design is expected to assist end-users in systematically working through the content, including through the key orientation questions, while also supporting them in discovering content that is relevant to the cancer challenge that they are facing, and other challenges that may be of relevant.

## Content should incorporate search engine optimization (SEO), especially for Google

Content should be designed to be accessible and discoverable directly through Google searching, in addition to any other internet media platforms. This recommendation reflects the common practices of the participants in the accompanying study for accessing online content as well as the fact that Google has been reported to have over 86% of current search engine market share [52]. In comparison to directly accessing content on well-known, reputable, or recommended websites via URL entry or bookmarks, browsing through Google results was identified as the primary strategy for identifying online content by the participants in the accompanying study.

While a complete discussion regarding search engine optimization (i.e., SEO) for Google [53, 54] is beyond the scope of this document, three key points are worth noting. First, Google’s indexing relies in part on keywords used in titles, headings, and hyperlinks [54]. Therefore, the page title, headings, and hyperlink descriptions should be both concise and describe the cancer challenge. When possible, use language in titles, headings, and text that includes terms likely to be used by the intended audience searching for content on the cancer challenge [54]. Engaging with patients and informal caregivers to understand what search terms and strategies they would use to find content on the cancer challenge being addressed is a key activity to understanding how to incorporate appropriate terms in titles, headings, and hyperlinks. Lastly, analysis of hyperlinks included in the page being indexed is an important part of Google’s indexing algorithm [54]. Including hyperlinks labeled with descriptive keywords to reputable content will help Google identify when it is appropriate to include the online content in search results [54].

**References**

46. Fleisher L, Bass S, Ruzek SB, McKeown-Conn N. Relationships among Internet health information use, patient behavior and self efficacy in newly diagnosed cancer patients who contact the National Cancer Institute's NCI Atlantic Region Cancer Information Service (CIS). Proc AMIA Symp. 2002:260-4. pmid: 12463827.

47. Eysenbach G. The impact of the Internet on cancer outcomes. CA Cancer J Clin. 2003;53(6):356-71. doi: 10.3322/canjclin.53.6.356. pmid: 15224975.

48. Helft PR, Hlubocky F, Daugherty CK. American oncologists' views of internet use by cancer patients: a mail survey of American Society of Clinical Oncology members. J Clin Oncol. 2003;21(5):942-7. doi: 10.1200/JCO.2003.08.007. pmid: 12610198.

49. Kelly B, Sloan D, Brown S, Seale J, Lauke P, Ball S, et al. Accessibility 2.0: Next Steps for Web Accessibility. Journal of Access Services. 2009;6(1-2):265-94. doi: 10.1080/15367960802301028.

50. Lewthwaite S. Web accessibility standards and disability: developing critical perspectives on accessibility. Disability and Rehabilitation. 2014;36(16):1375-83. doi: 10.3109/09638288.2014.938178.

51. Silberg WM, Lundberg GD, Musacchio RA. Assessing, Controlling, and Assuring the Quality of Medical Information on the Internet: Caveant Lector et Viewor—Let the Reader and Viewer Beware. JAMA. 1997;277(15):1244-5. doi: 10.1001/jama.1997.03540390074039.

52. Davies D. Meet the 7 Most Popular Search Engines in the World: Search Engine Journal; 2021. Available from: <https://www.searchenginejournal.com/seo-guide/meet-search-engines/#close>.

53. Cushman M. Search engine optimization: What is it and why should we care? Res Pract Thromb Haemost. 2018;2(2):180-1. doi: 10.1002/rth2.12098. pmid: 30046718.

54. Google. Search Engine Optimization (SEO) Starter Guide 2023. Available from: <https://developers.google.com/search/docs/fundamentals/seo-starter-guide>.
