# Supplementary material for: Navigating the Cancer Journey Using Web-Based Information: Grounded Theory Emerging From the Lived Experience of Cancer Patients and Informal Caregivers With Implications for Web-Based Content Design
Source: JMIR Cancer. 2023 May 17;9:e41740. doi: 10.2196/41740 (PMC10233434; doi:10.2196/41740)
Supplement: Multimedia Appendix 8 [file cancer_v9i1e41740_app8.docx]

# Exploring the sample content: Orientation theory in action

As part of the study described in the accompanying manuscript, sample content was created. This process of content creation was embedded in the methods that resulted in the emergence of orientation theory. After initial theory development, and the identification of a relevant topic for content creation in collaboration with the participants, an initial draft of the content was developed by the researcher (MT) based on the emerging theoretical concepts. Next, the content was reviewed with the study participants through interviews and focus groups, with the data collected from these study activities being analyzed to guide further development of the emerging theory. In turn, the revised theory informed further development of the sample content, which was subsequently re-reviewed with the participants. This process repeated iteratively until no further revision to the theory were needed as the sample content were identified as well laid out, useful, and complete by the study participants. Importantly, the recommendations described in the main manuscript and elaborated on Multimedia Appendix 7 were informed in large part by the iterative process of data analysis and collection that resulted in the sample content.

The sample content is currently located online [55]. The following sections provides a top to bottom review of the sample content with commentary informed by orientation theory and the five recommendations forwarded in the manuscript and Multimedia Appendix 7. Of note, the following sections do not specifically address how the content is informed by recommendations for search engine optimization (SEO) for Google (i.e., recommendation #5, Multimedia Appendix 7). However, it should be noted, that the use of short, concise, descriptive, titles, headers, and hyperlinks will assist with SEO. Additionally, the inclusion of hyperlinks for both similar and related content are also expected to assist with Google SEO.

## Content panel #1: Orientation panel


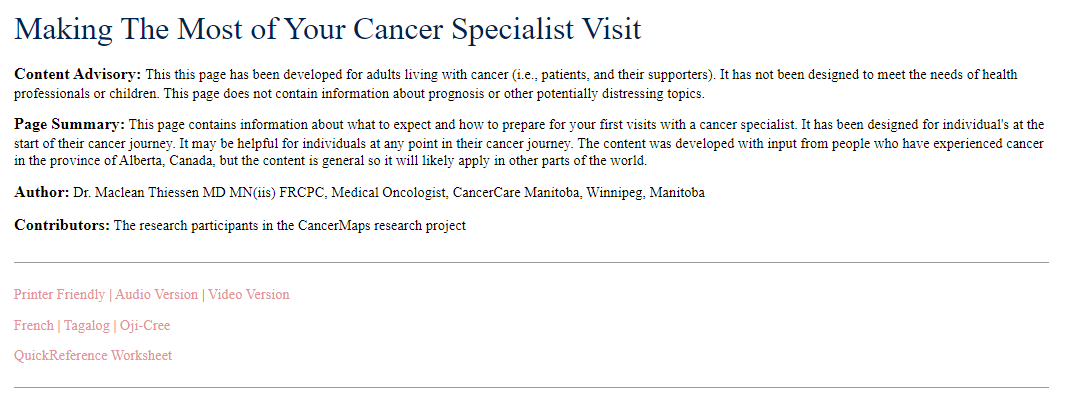


The orientation panel is positioned at the top of the sample content and provides essential information for the end-user about the content that is to follow. The first three elements orient the end-user to what is being addressed in the content. First, a title makes clear reference to the cancer challenge being addressed (i.e., meeting with a cancer specialist). Second, a content advisory informs the end-user about any potentially sensitive information that may be contained below so. Finally, the page summary provides additional details to the end-user about what the content covers, and who it is specifically designed for. These three elements are designed to help the end-user become quickly oriented to the content provided and decide whether they wish to invest further time and energy into navigating it. This first section primarily reflects the principles outlined in recommendation #1, Multimedia Appendix 7.

Next, author and contributor information are included. This content helps end-users who are comparing multiple sources of information make determinations regarding the validity/accuracy of any specific content, especially when information that is inconsistent with that from other sources is identified. Including this reflects recommendation #3, Multimedia Appendix 7. This is followed by links to alternate versions of the content. These alternate versions reflect that the heterogenous personal characteristics of individuals living with cancer result in different needs in terms of content format. Overall, the orientation panel is a key piece of the content, in that it helps orient the end-user to what is included in the following content, how alternative formats can be accessed, and what level of trust they should put in the content.

## Content panel #2: Addressing the why and what to expect


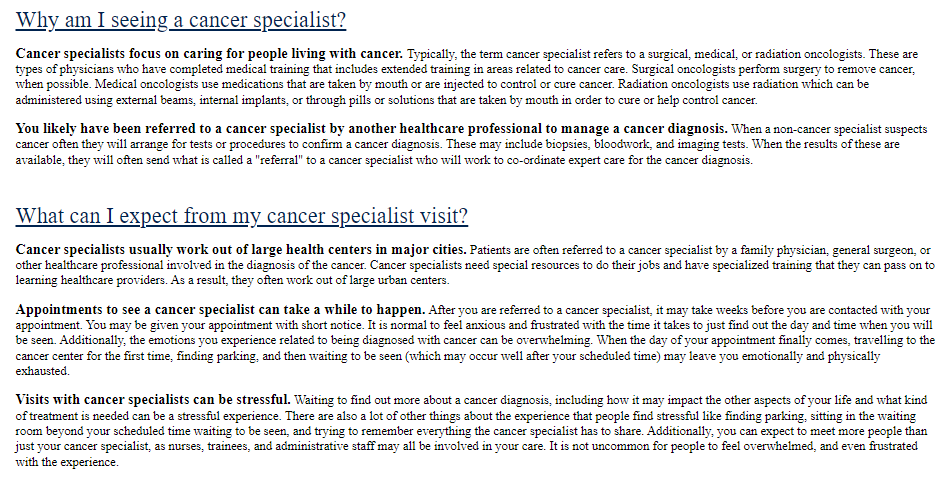


Orientation theory identifies that, with regards to any specific cancer challenge, there are essentially three key orientation questions that individuals search out answers for: 1) *why is this happening*; 2) *what can I expect*; and 3) *what can I do*. In the first section of the body of the sample content the first two key orientation questions are addressed. Headers are used to identity which orientation question is being answered, with each paragraph addressing a sub-question. Importantly, for this content, the sub-questions were identified based on the data collected from the participants about what they experienced regarding the specific cancer challenge of meeting with their oncologist.

This approach reflects recommendations #1 and #3 (Multimedia Appendix 7) as the key orientation questions for the cancer challenge are addressed systematically. Additionally, the approach of basing the content on the experience of those that have experienced speaks to why including multiple perspectives in content creation can enhance the quality of the content. Without the input from the participants, it may have been possible for the author to identify some of the possible sub-questions, however, there were some that were not predicted a priori by the author as important but were highlighted by the participants. Conversely, the author was able to provide details that added to the study participants understanding of the cancer challenge by providing additional insights from the perspective of a healthcare professional.

## Content panel #3: Addressing what can I do


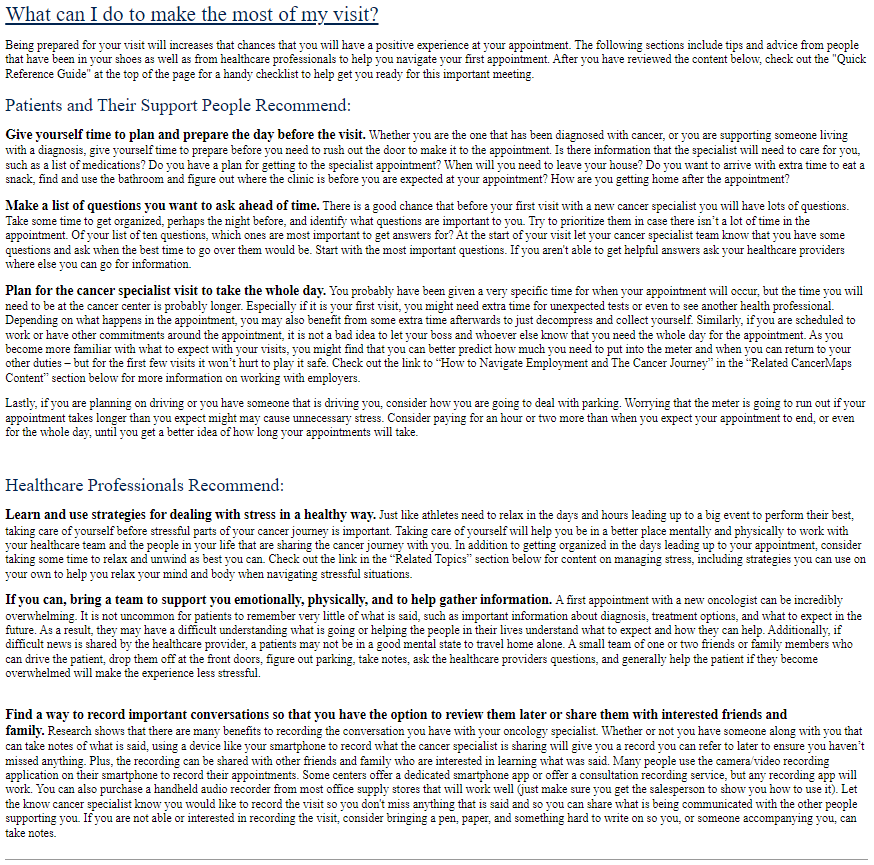


After the first two key orientation questions have been addressed, the body of the content moves to address the third orientation question. This section outlines what actions the individual can take to improve their outcome with the cancer challenge. The inclusion of this section reflects the importance of addressing all three key orientation questions in content tackling a cancer challenge. Without it, individuals may be left wondering what they can do, or unknowingly taking actions that may make their experience worse. This again reflects recommendation #1 (Multimedia Appendix 7) and the importance of ensuring that all key orientation questions are systematically addressed.

Additionally, this section also is divided into what the participants in the study recommended and what healthcare professionals recommend in terms of making the most of a cancer specialist visit. Here again, the different perspectives that individuals have to a specific cancer challenge is important and highlighted. The recommendations generated by the participants in the orientation theory study are very different from those that came directly from the author, who is a practicing medical oncologist. Additionally, this speaks to the importance of clearly identifying who informed the content, in keeping with recommendation #3 (Multimedia Appendix 7) as end-users my wonder why recommendations for navigating cancer specialist visits differ from other sources [56].

## Content panel #4: Hyperlinks


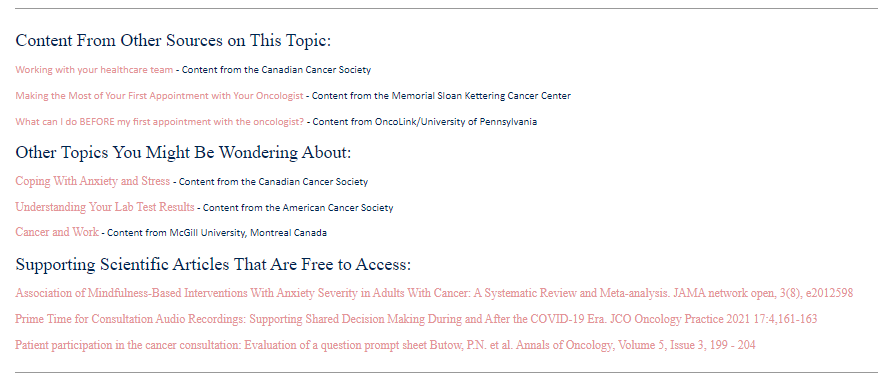


The placement and content of hyperlinks in the sample content reflects recommendation #4 (Multimedia Appendix 7). Regarding placement, orientation theory identifies that one of the challenges with internet content is that hyperlinks can be distracting and direct individuals away from content before all the key orientation questions have been addressed. Therefore, to avoid this, hyperlinks should be placed after the key orientation questions have been addressed.

Regarding the specific links, it was identified in the study that resulted in orientation theory that the participants often verify the information they come across, including that from the internet and from healthcare professionals, by reviewing content from multiple sources. Therefore, the sample content includes links to similar material from other sources, in an effort to connect the end-user with content from other sources so they can verify for themselves what is accurate and obtain additional perspective. Lastly, as individuals face multiple cancer challenges at once, hyperlinks are included for other cancer challenges they may be facing at the time of their first visits with the cancer specialist.

The links to peer-reviewed articles that are relevant to the cancer challenge is in keeping with concepts described by orientation theory. Some participants expressed a preference for reading (or even simply accessing), relevant scientific peer-reviewed articles for the cancer challenges they were facing. This reflects both the unique personal characteristics of individuals informing what formats are useful (e.g., recommendation #2, Multimedia Appendix 7), as well as the importance of credibility in helping individuals trust the information they are encountering in the orientation process.

## Content panel #5: How to find a real person


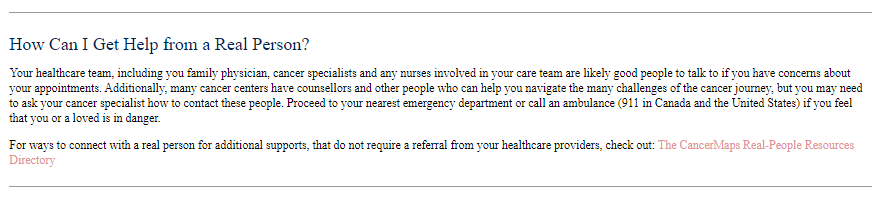


It was identified in the orientation theory study that the internet is often accessed when individuals cannot access their healthcare team, or, alternatively, are uncomfortable asking a member of their healthcare team for information about the cancer challenge they are facing. These issues did not always reflect a preference for internet information, and it was not uncommon for the participants to express that they would have preferred to have been able to access a healthcare provider that they were confident and comfortable asking their question to, in a timely manner. Therefore, this panel works to address the issues by providing end-users with access to resources where they can find additional information about their cancer challenge or other challenges through interaction interacting with a real person. Like the text addressing the key orientation question of *what can I do* (see: Content panel #4), this section provides the end-user with another option in terms of managing their cancer challenges.

## Content Panel #6: Publishing information


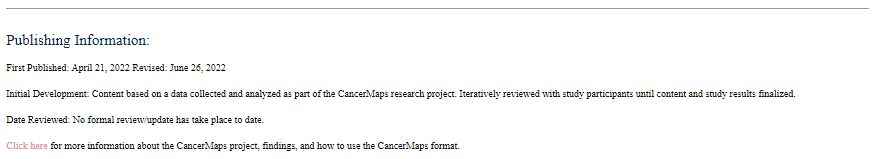


Lastly, this panel includes information about initial publication dates, time of updates and more details about how the content was developed. Publication dates, and dates of review were identified by some participants as important, but not as consistently as the other elements of the content and were unable to be clearly linked to orientation theory. The additional details about how the content was developed, were endorsed as adding some value by a few participants, and were initially in the first content panel. However, they were subsequently placed at the bottom of the page to make the first content panel more concise in keeping with participant feedback.

**References**

55. Sample Content: Preparing For Meetings With Cancer Specialists 2022 [cited 2022 June 29]. Available from: <http://sample.cancermaps.ca>.

56. What to Expect in Your First Meeting with Your Cancer Doctor | Dana-Farber. 2019.
